# Supplementary material for: A high-resolution mRNA expression time course of embryonic development in zebrafish
Source: eLife. 2017 Nov 16;6:e30860. doi: 10.7554/eLife.30860 (PMC5690287; doi:10.7554/eLife.30860)
Supplement: Supplementary file 6. [file elife-30860-supp6.zip › biolayout-clusters-files/Cluster087-genes.html]

Cluster087


# Cluster087: Genes

| Ensembl ID | Gene Name | Chr | Start | End | Biotype |
| --- | --- | --- | --- | --- | --- |
| ENSDARG00000059257 | RCVRN (1 of many) | 12 | 310187 | 314209 | protein\_coding |
| ENSDARG00000007219 | actn1 | 13 | 36195256 | 36265670 | protein\_coding |
| ENSDARG00000103747 | cav1 | 25 | 18467076 | 18481186 | protein\_coding |
| ENSDARG00000036616 | gpr182 | 23 | 36530893 | 36533179 | protein\_coding |
| ENSDARG00000044074 | loxl2b | 5 | 26840168 | 26887645 | protein\_coding |
| ENSDARG00000037278 | lrata | 1 | 25005969 | 25007210 | protein\_coding |
| ENSDARG00000074815 | mxra5a | 1 | 32589529 | 32600676 | protein\_coding |
| ENSDARG00000019096 | myl7 | 8 | 40417522 | 40425350 | protein\_coding |
| ENSDARG00000079570 | rspo2 | 16 | 38504338 | 38604289 | protein\_coding |
| ENSDARG00000030967 | si:ch211-51a6.2 | 13 | 21649329 | 21691481 | protein\_coding |
| ENSDARG00000102056 | si:dkeyp-94b4.1 | 12 | 15648077 | 15780684 | protein\_coding |
| ENSDARG00000011400 | tnnc1a | 23 | 4955108 | 4960291 | protein\_coding |
| ENSDARG00000075441 | vwa2 | 12 | 30197406 | 30230542 | protein\_coding |
| ENSDARG00000041339 | zgc:92380 | 9 | 48309686 | 48342569 | protein\_coding |
